# Ultrafast spontaneous emission source using plasmonic nanoantennas

**DOI:** 10.1038/ncomms8788

**Published:** 2015-07-27

**Authors:** Thang B. Hoang, Gleb M. Akselrod, Christos Argyropoulos, Jiani Huang, David R. Smith, Maiken H. Mikkelsen

**Affiliations:** 1 Department of Physics, Duke University, Durham, North Carolina 27708, USA.; 2 Center for Metamaterials and Integrated Plasmonics, Duke University, Durham, North Carolina 27708, USA.; 3 Department of Electrical and Computer Engineering, Duke University, Durham, North Carolina 27708, USA.

## Abstract

Typical emitters such as molecules, quantum dots and semiconductor quantum wells have slow spontaneous emission with lifetimes of 1–10 ns, creating a mismatch with high-speed nanoscale optoelectronic devices such as light-emitting diodes, single-photon sources and lasers. Here we experimentally demonstrate an ultrafast (<11 ps) yet efficient source of spontaneous emission, corresponding to an emission rate exceeding 90 GHz, using a hybrid structure of single plasmonic nanopatch antennas coupled to colloidal quantum dots. The antennas consist of silver nanocubes coupled to a gold film separated by a thin polymer spacer layer and colloidal core–shell quantum dots, a stable and technologically relevant emitter. We show an increase in the spontaneous emission rate of a factor of 880 and simultaneously a 2,300-fold enhancement in the total fluorescence intensity, which indicates a high radiative quantum efficiency of ∼50%. The nanopatch antenna geometry can be tuned from the visible to the near infrared, providing a promising approach for nanophotonics based on ultrafast spontaneous emission.

## Abstract (web summary)

Typical emitters such as molecules and quantum dots have slow spontaneous emission with lifetimes of 1–10 ns. Here, Hoang *et al*. have fabricated a hybrid structure of plasmonic nanopatch antennas coupled to quantum dots, achieving ultrafast spontaneous emission with a lifetime of 11 ps.

## Graphical abstract

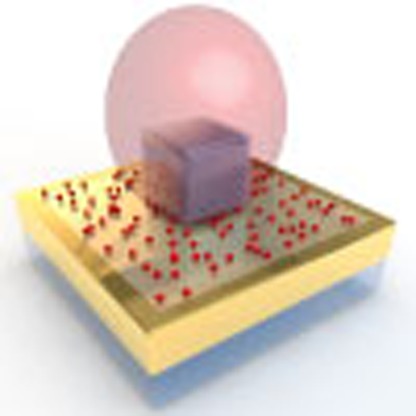

Spontaneous emission is the process of photon emission by a quantum system as it transitions from an excited state to a ground state. The excited state lifetime is determined by the spatial overlap between the excited and ground state wavefunctions, and the photonic density of states that is seen by the emitter. In quantum systems used as sources of spontaneous emission—such as molecules, quantum dots (QDs) and semiconductor quantum wells—this lifetime is typically on the scale of 1–10 ns, corresponding to emission rates of 100–1,000 MHz. This relatively slow rate of spontaneous emission is limited both by the small physical size of the emitters and the low photonic density of states of free space. For photonic devices that are based on light emission, these long radiative lifetimes are a hindrance to high-speed devices.

A spontaneous emission source of particular interest for device applications is semiconductor QDs. These emitters combine a tuneable emission wavelength at room temperature, high radiative quantum efficiency, excellent photostability and ease of integration with other materials^1^. For example, colloidal QDs have been demonstrated as stable, room temperature single-photon sources^2^,^3^, but the slow radiative rate associated with these systems limits the attainable repetition rate. Likewise, light-emitting diodes (LEDs) are often not used in telecommunications, in part, due to the long spontaneous emission lifetimes. QDs are also promising as gain media for micro- and nanoscale lasers, but achieving a low lasing threshold has proven challenging due to non-radiative Auger recombination outcompeting the slow intrinsic radiative lifetime of ∼20 ns (refs 4, 5).

To increase the rate of spontaneous emission of QDs, a number of approaches have been developed to engineer the photonic environment of the emitter and increase the photonic density of states. The figure of merit that characterizes the enhancement in the spontaneous emission rate is the Purcell factor^6^, 
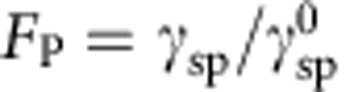
, where 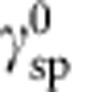 is the intrinsic spontaneous emission rate and *γ*_sp_ is the enhanced rate. Dielectric cavities coupled to epitaxial QDs were first used for Purcell enhancement and improved emission directionality^7^. However, obtaining large Purcell factors, *F*_P_ (∼*Q*/*V*), in dielectric cavities demands high-quality (*Q*) factors and small mode volumes (*V*)^8^,^9^. Earlier work by Hennessy *et al*.^10^ showed that significant fabrication effort is required to position a single QD at the maximum field of the cavity and to spectrally tune the QD emission to match the cavity mode. Despite advanced techniques to fabricate and tune high-Q cavities, including micropillar cavities^11^,^12^,^13^, microtoroid resonators^14^ and photonic crystal cavities^15^,^16^,^17^,^18^, experimental values of the Purcell factor in dielectric optical cavities are presently limited to ∼75 (refs 19, 20, 21, 22). In addition, these typically narrow band systems often require low temperatures and are not well suited for tailoring the broadband emission from room temperature emitters. However, room temperature modulation of an LED based on a photonic crystal cavity has shown modulation rates of up to 10 GHz (ref. 23).

Plasmonic nanocavities, such as bowties^24^, dimers^25^ and film-coupled nanoparticles^26^,^27^,^28^, have attracted interest in recent years because they offer large field enhancements, broad resonances (typical *Q* factors ∼10–30), room temperature operation and, in some cases, can be easily fabricated via colloidal synthesis. Plasmonic nanocavities support strong field enhancements and a strongly modified photonic density of states, thus providing a flexible means of controlling the spontaneous emission rate of quantum emitters and other light–matter interactions at the nanoscale^24^,^29^,^30^,^31^,^32^,^33^. Typical drawbacks of plasmonics include losses due to non-radiative decay in the metals and limited control over the directionality of emission. Various plasmonic structures have been utilized to enhance the emission of QDs, but so far only limited Purcell factors of <145 have been demonstrated^25^,^26^,^28^,^33^,^34^. Higher Purcell factors of up to 1,000 have been obtained for molecules^27^,^36^,^35^, but such large enhancements of QDs have so far proven elusive. Furthermore, in plasmonic structures, the Purcell enhancements are typically accompanied by low radiative efficiency due to significant non-radiative losses^28^,^29^,^33^, or have low directionality of emission^29^. For example, hybrid QD and Au nanoparticle structures assembled by atomic force microscopy nanomanipulation have shown Purcell factors up to 145 but radiative decay rate enhancements of only ∼8 (ref. 33). One-dimensional metamaterials with a hyperbolic dispersion have also been used to achieve control of spontaneous emission, but the Purcell factors have been limited to ∼10 (ref. 36).

In this work, we utilize plasmonic nanopatch antennas (NPAs) to experimentally demonstrate an ultrafast and efficient source of spontaneous emission with a lifetime shorter than 11 ps, limited by the detector resolution, corresponding to an emission rate faster than 90 GHz. The ultrafast emission is achieved by integrating colloidal and photostable semiconductor QDs into the plasmonic structure. Nanometre precision control of the antenna dimensions and a quantitative understanding of the emission rates enables us to achieve Purcell factors of up to *F*_p_=880, while maintaining a high radiative efficiency and directional emission.

## Results

### NPA system

**Figure 1:**
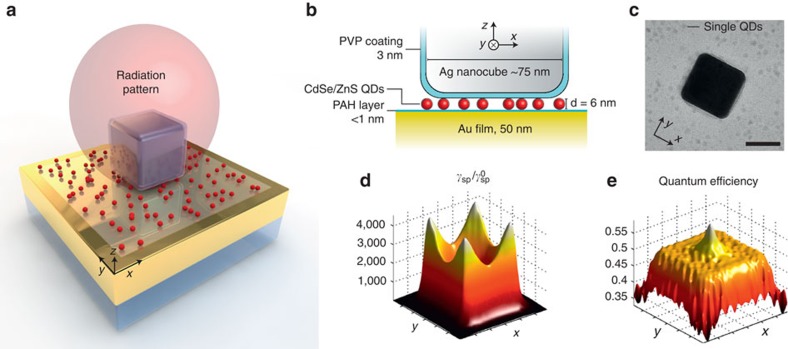
QDs coupled to plasmonic NPAs. (**a**) Three-dimensional illustration of a NPA. The simulated directional radiation pattern from the antenna is shown in red. (**b**) Cross-sectional schematic of the NPA consisting of a silver nanocube on top of a Au film, separated by a 1 nm polyelectrolyte spacer layer and a sparse layer of ∼6 nm diameter CdSe/ZnS QDs. (**c**) Transmission electron microscopy image of a silver nanocube and QDs; scale bar, 50 nm. (**d**,**e**) Simulated spatial maps of (**d**) spontaneous emission rate enhancement (Purcell factor) and (**e**) radiative quantum efficiency for a vertically oriented QD dipole situated in the gap between the nanocube and the Au film.

The NPA system consists of a silver nanocube coupled to a metal film, separated by a controlled nanoscale (∼10 nm) dielectric spacer layer and colloidal QDs (Fig. 1a–c). The fundamental plasmonic mode is a Fabry–Pérot resonance resulting from multiple reflections of the waveguide mode beneath the nanocube that propagates within the gap region. The dominant field is normal to the gap with the maximum field enhancement occurring near the nanocube edges and corners^37^,^38^.

This unique plasmonic mode has three key advantages: (i) the resonance wavelength can be tuned by adjusting the nanocube size or the nanogap thickness while maintaining large field enhancements of up to 200-fold^37^. Using full-wave simulations, we predict that these field enhancements translate into large Purcell factors, 
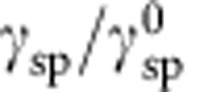
, of up to 4,000 for emitters embedded in the gap region (Fig. 1d). The largest enhancements occur for emitters located near the corners of the nanocube and with transition dipoles oriented vertically (along the *z* axis), in the direction of the electric fields associated with the fundamental plasmon mode. (ii) Even with small plasmonic gaps (<10 nm) and large field enhancements, the radiative efficiency of emitters coupled to the plasmon mode remains high (∼50%; Fig. 1e). (iii) The NPAs exhibit a highly directional radiation pattern (Fig. 1a) with a collection efficiency of 84% using a 0.9 NA objective^39^. Figure 1a shows a simulated far-field radiation pattern, assuming that a dipole is pointing in the vertical direction and is located near the nanocube corner, where the largest field enhancement can be achieved.

**Figure 2:**
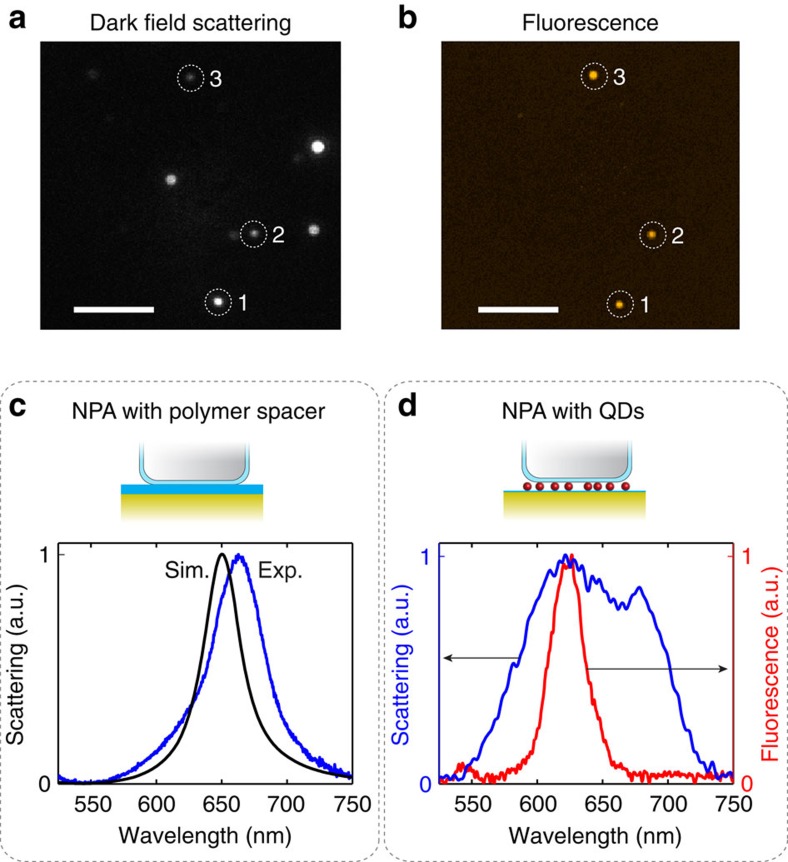
Spectral properties of the NPA. (**a**) Dark field scattering image showing individual NPAs as bright spots with different intensities because of different scattering amplitudes and resonant wavelengths. Scale bar, 5 μm. (**b**) Fluorescence image of the same location when illuminated by a defocused 514 nm CW laser. Several NPAs, labelled 1, 2 and 3, are visible in the scattering and fluorescence images. Only NPAs resonant with the QD emission are visible in the fluorescence image. Scale bar, 5 μm. (**c**) Measured and simulated scattering spectrum of a single NPA with a polymer-filled gap and no QDs, in normalized units. (**d**) Measured scattering spectrum of a single NPA containing QDs in the gap region. The measured fluorescence spectrum for QDs coupled to the NPA is also displayed in red showing good overlap with the scattering spectrum. Exp., experimental; Sim., simulation.

The NPAs are fabricated via the deposition of a sparse layer of colloidal QDs on top of a ∼1 nm poly(allylamine) hydrochloride (PAH) layer on a gold (Au) film, followed by electrostatic adhesion of colloidally synthesized silver nanocubes. On average, ∼10 QDs are located under each nanocube, as determined by transmission electron microscopy of a similar sample prepared on a carbon film instead of a Au film (Fig. 1c). Using a custom-built microscope, individual NPAs are identified by dark-field and fluorescence imaging (Fig. 2a,b), followed by spectroscopy (Fig. 2c,d) and time-resolved fluorescence measurements on the located nanoparticles (see Methods). Owing to the distribution of nanocube sizes, only a subset of NPAs is resonant with the QD fluorescence, as shown in Fig. 2b.

In the absence of QDs in the nanogap region, the scattering spectrum of a single NPA is dominated by the lowest-order fundamental mode with a single Lorentz peak as observed in experiments (Fig. 2c). Full-wave simulations show a similar resonance peak (Fig. 2c) with the resonance wavelength determined by the nanocube size, and by the thickness and dielectric constant of the material in the gap region. When QDs are present in the nanogap, the scattering spectrum is broadened (Fig. 2d). Similar broadening, with some variation, is observed from the other measured NPAs (Supplementary Fig. 1). This mode broadening is attributed to an inhomogenenous dielectric environment in the cavity. The random spatial distribution of the QD positions results in a non-uniform dielectric constant in the nanogap and may also cause the nanocube to be tilted relative to the Au film. These geometrical effects can cause symmetry breaking between the TM and TE excitation modes, resulting in broadening of the scattering spectrum^37^. Strong coupling can be ruled out as a mechanism for the mode splitting based on an estimate of the ensemble QD-nanocavity coupling energy (22 meV), which is found to be significantly smaller than the linewidth of the cavity resonance (150 meV; Supplementary Discussion). Furthermore, the mode splitting does not consistently occur around the energy of the QD emission peak, but instead varies between NPAs (Supplementary Fig. 1). In addition, the emission spectrum of the QDs coupled to the NPA is unchanged relative to the intrinsic QD spectrum, confirming that the system is in the weak coupling regime (Supplementary Fig. 2).

### Enhancement of time-integrated fluorescence

**Figure 3:**
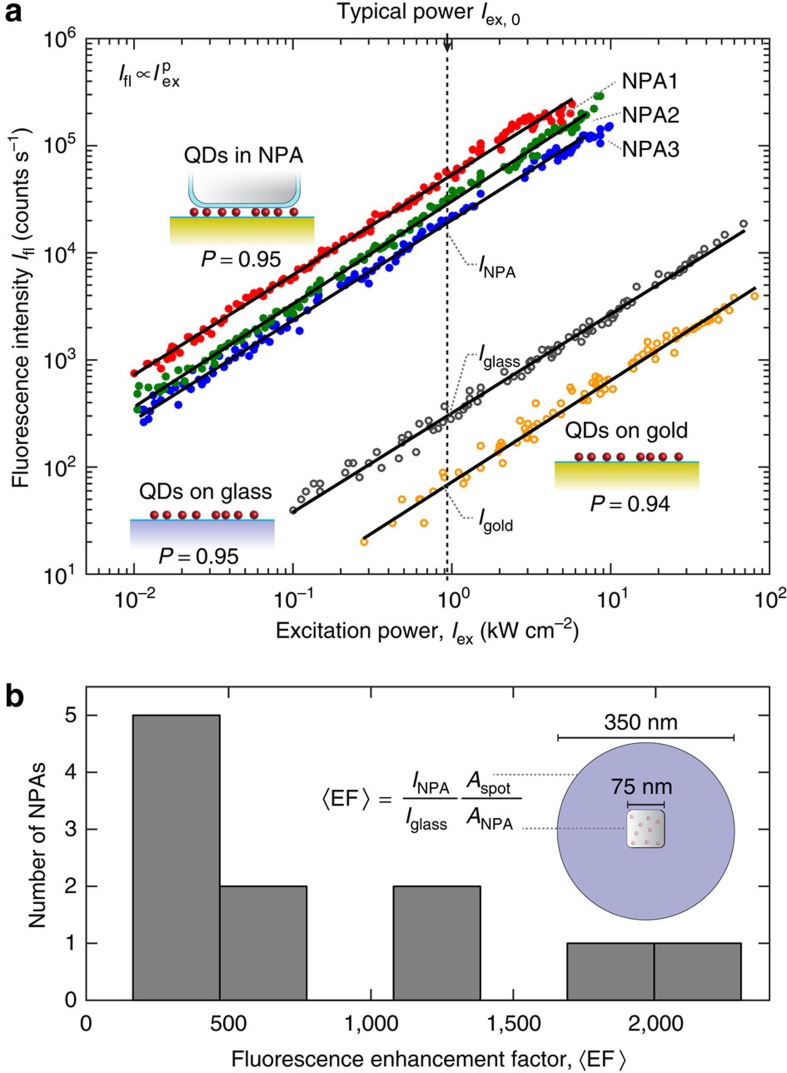
Fluorescence enhancement of QDs coupled to NPAs. (**a**) QD fluorescence intensity as a function of average incident laser power in three cases—on a glass slide, on a Au film and coupled to individual NPAs (NPAs 1–3). The solid lines are fits to a power law, with the power exponent, *p*, showing a nearly linear scaling. The vertical dashed line indicates the power at which subsequent measurements in this paper are performed under pulsed excitation. (**b**) Histogram showing the distribution of the fluorescence enhancement factors of the 11 measured NPAs.

To quantitatively estimate the enhancement in the fluorescence intensity of the QDs coupled to a single plasmonic NPA, we conducted a series of experiments on three different samples: (i) a sample containing QDs coupled to NPAs as described above; (ii) a sample of QDs adhered on top of a PAH layer on a Au film but without any nanocubes; and (iii) a sample with QDs adhered to a PAH layer on a glass slide. To ensure the same surface density of QDs, all samples are prepared with the same concentration of QDs in solution and had the same surface chemistry (PAH) before spin coating the QDs. For excitation, a 535-nm Ti:sapphire laser is used with a pulse length of ∼150 fs, which is passed through a pulse picker to reduce the repetition rate from 80 to 40 MHz. The excitation laser is focused to a diffraction-limited spot, ∼300 nm in diameter and the QD fluorescence is collected in an epifluorescence configuration and measured by an avalanche photodiode (see Methods). Figure 3a shows the dependence of the QD fluorescence intensity on the laser excitation power for the three samples described above. The fluorescence intensity from the QDs coupled to a single NPA is substantially higher than from the QDs on a PAH layer on a glass slide or on a Au film. For QDs on a PAH layer on a Au film without any nanocubes, we find that the fluorescence is quenched by ∼70% compared with QDs on glass. This quenching is attributed to short-range non-radiative energy transfer between the QDs and the Au film^40^,^41^. The emission intensity from QDs coupled to a single NPA shows linear scaling with excitation power density in the range of 0.01–10 kW cm^−2^. At higher excitation power densities, permanent photobleaching of the QDs occurs before saturation of the excited state population can be reached. All subsequent measurements in this paper are conducted at an excitation power density of *I*_ex,0_=1 kW cm^−2^. For structures with a polymer gap layer and no QDs, we found that the NPA scattering resonance was unmodified and stable for average excitation power densities with the femtosecond laser of up to 10 MW cm^−2^.

The average enhancement in fluorescence intensity due to the NPA is given by the fluorescence enhancement factor


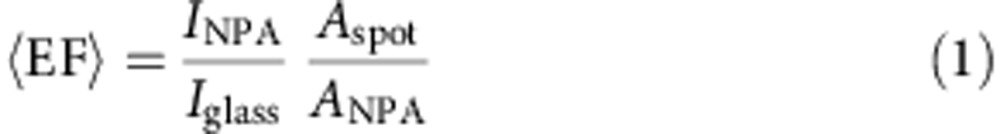


where *I*_NPA_ and *I*_glass_ are the fluorescence intensities measured in the far field from a ∼300 nm diameter laser spot exciting a single NPA and a glass slide with QDs, respectively. Both the intensities were corrected for background fluorescence around the nanocube and detector dark counts. The intensities are normalized by the area from which the fluorescence originates in each measurement, where *A*_spot_ is the area of the excitation spot and *A*_NPA_ is the area under a single nanocube. The relationship between the size of the nanocubes and resonance wavelength for a given gap thickness has been established previously^37^. In addition, we independently verified using scanning electron microscopy that, for example, NPAs with a resonance of ∼635 nm indeed correspond to a lateral nanocube size of 75 nm (Supplementary Fig. 3). Measurements of the fluorescence intensity from 11 individual NPAs show enhancement factors that vary from 177 to 2,300, with an average value of 〈EF〉_avg_=831 (Fig. 3b). For these measurements, we selected only NPAs with plasmon resonances around the QD emission wavelength (625–635 nm). The variation in 〈EF〉 is attributed to two factors: (i) the random spatial distribution of QDs within the nanogap, with QDs near the nanocube edges experiencing higher excitation field enhancements^37^,^38^,^42^, (Fig. 1d) and (ii) the random orientation of each QD^39^, with absorption dipoles oriented vertically having the largest coupling to the excitation field.

Before investigating the emission rate enhancement, it is crucial to establish the origin of the total fluorescence enhancement and the radiative quantum efficiency of the QDs coupled to the NPA. The measured fluorescence enhancement is a combination of enhancements in the collection efficiency, the excitation rate and the radiative quantum efficiency that is averaged over all positions and orientations of the QDs in the nanogap:


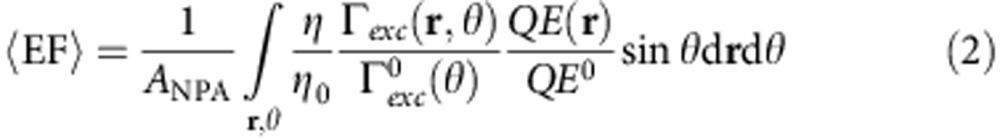


where *η*, Γ and *QE* are the emission collection efficiency, excitation rate and quantum efficiency in the NPA sample, respectively. Each of these values is normalized by the same quantity corresponding to the QDs on glass, which is denoted ‘0'. The excitation rate Γ_exc_ depends both on the location of the QD in the nanogap **r** and the dipole orientation *θ* (ref. 39). The excitation rate term is


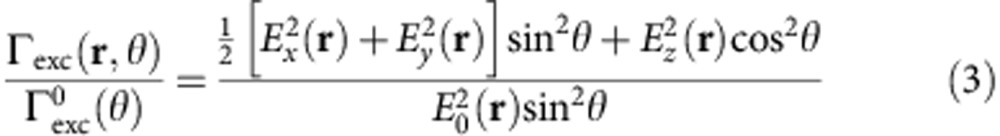


Where *E*(**r**)_*x,y,z*_ are the electric field components in the *x*, *y* and *z* directions at the excitation frequency (Supplementary Fig. 4). The incident electric field for the control sample is assumed to be in-plane. Given a random distribution of QD orientations, integrating equation (3) over *θ* and *r* yields a value of the average excitation rate enhancement of 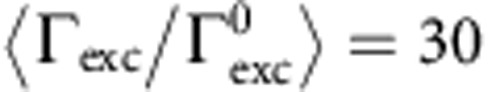. Using the simulated far-field radiation pattern (Fig. 1a), we estimated a collection efficiency from the NPA of *η*=84% using an objective with NA=0.9 (ref. 39). Meanwhile, the collection efficiency from randomly oriented QDs on the glass slide using the same NA objective was estimated to be *η*_0_=19% (ref. 43). The QE in the NPA is approximately spatially uniform in the gap region (Fig. 1e) with an average value of ∼0.5, whereas the intrinsic quantum yield of the QDs in solid state is taken to be QE_0_=0.1. By combining these calculations, the average fluorescence enhancement factor from the simulations is 〈EF〉=660. The agreement in the fluorescence enhancement factor between the theory and the experiment indicates that the high radiative quantum efficiency of the NPA predicted by the simulations is accurate.

### Enhancement in spontaneous emission rate

**Figure 4:**
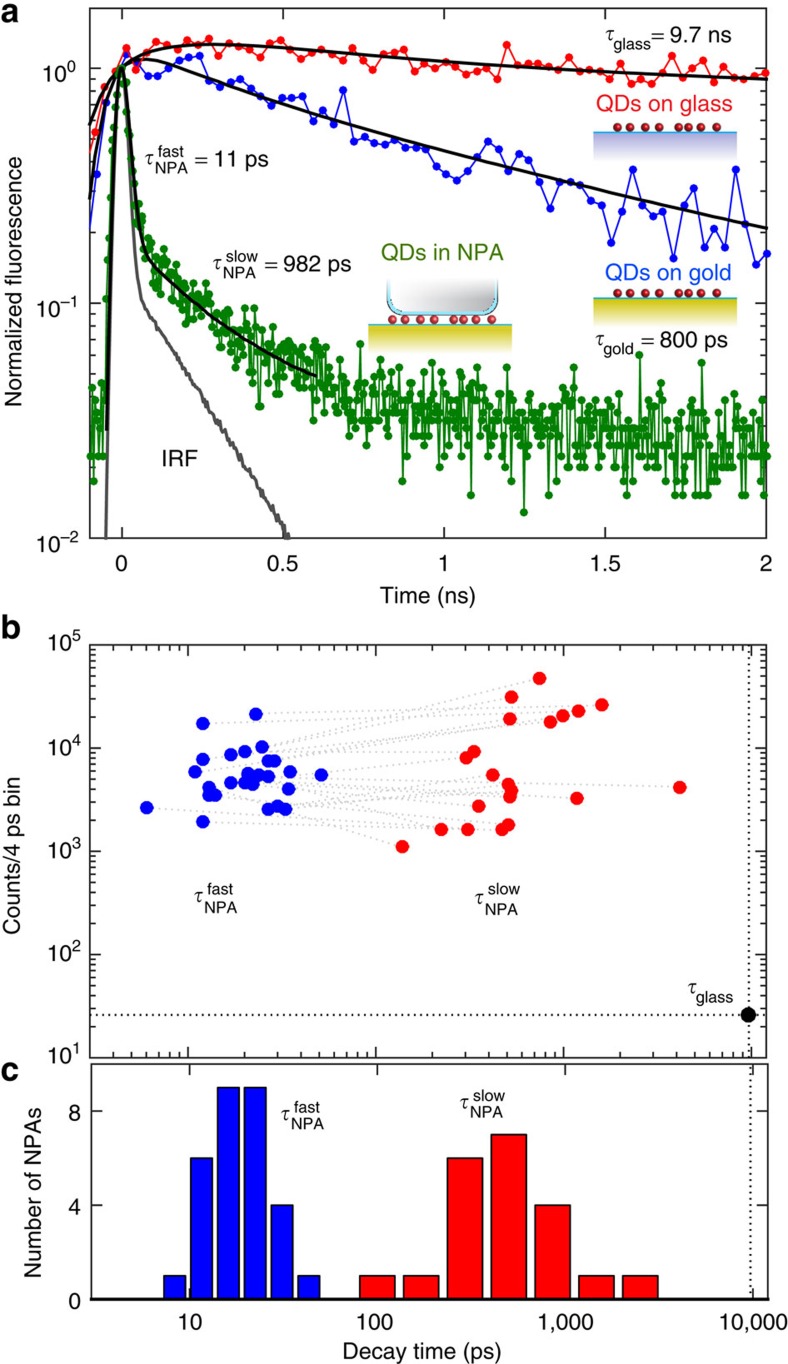
Increased spontaneous emission rate of QDs coupled to NPAs. (**a**) Normalized time-resolved fluorescence of QDs on a glass slide (red) compared with QDs on a Au film (blue) and coupled to a single NPA (green). The instrument response function (IRF) is also shown^33^. Fits to exponential functions convolved with the IRF are shown in black. A single exponential function is used for the QDs on glass and Au. A biexponential function is used to fit the NPA decay. (**b**) Scatter plot of fluorescence decay times for ∼30 NPAs showing the relative intensity contributions of the fast and slow decay components. The dashed line connects the two components for each individual NPA. Some decay curves show a more robust fit to a single exponential, and, in these cases, the slow component is not shown. (**c**) A histogram showing the decay time distribution of the fast and slow components of the ∼30 individually measured NPAs.

Having established the high QE of QDs coupled to single NPAs, we turn to time-resolved fluorescence measurements to demonstrate the enhancement of the spontaneous emission rate. Figure 4a shows the normalized time dependence of the emission of QDs on glass, on a Au film, and coupled to a single NPA. The decay of QDs on a glass slide features a single exponential component with a lifetime of *τ*_glass_=9.7±0.1 ns. On a Au film without nanocubes, the QDs show a shortened lifetime of *τ*_gold_=0.8±0.03 ns but with a significantly reduced intensity, as shown in Fig. 3a, which is the result of direct metal quenching^24^,^42^.

When the QDs are coupled to the NPA, a dramatic decrease in the fluorescence lifetime is observed (Fig. 4a). This decrease in lifetime is accompanied by a simultaneous increase in the time-integrated fluorescence (Fig. 3a). The measured fluorescence from a single NPA is a summation of the emissions from all of the random lateral positions and orientations of QDs in the gap region, as each of these QDs has a different emission rate. The random dipole orientation of QDs ensures that a subset of QDs is always optimally coupled to the NPA, unlike horizontally oriented organic molecules, which have unfavourable coupling to the dominant vertical electric field^39^. The spatial and orientational summation is expected to produce a non-exponential decay curve with a distribution of rates^39^. However, the observed fluorescence decay approaches the instrument response function (IRF) of the detector (∼30 ps full width at half maximum), which hinders extraction of the full-rate distribution. Instead, we use a biexponential function deconvolved with the instrument response as an approximate fitting model, resulting in time constants of 
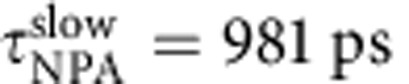
 and 
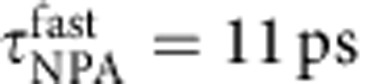
 for the NPA shown in Fig. 4a. The dominant fast component corresponds to a Purcell enhancement relative to QDs on glass of 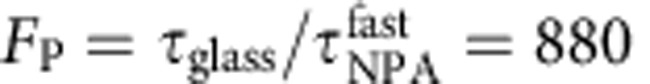. We note that this Purcell factor represents only a lower bound due to the detector resolution limit. The relative amplitudes of the fast and slow fluorescence decay components for ∼30 other NPAs are shown in Fig. 4b along with the decay lifetime distribution in Fig. 4c. For some fluorescence decay curves, a single exponential was found to be a more robust fitting function because the lifetimes approached the IRF (Fig. 4b). All of the NPAs showed a fast decay component in the range of 11–51 ps that corresponds to a maximum Purcell factor of 190 to 880. Critically, these large Purcell enhancements are obtained simultaneously with a high radiative QE, as demonstrated above.

## Discussion

To determine the Purcell enhancement using simulations, we average the position of the dipole source under the nanocube (Fig. 1d) and average over all dipole orientations because the QDs are expected to be randomly oriented. This results in a predicted Purcell factor of *F*_P_=990, corresponding to a lifetime of 10 ps, which is only slightly above the range of the measured Purcell factors. This result suggests that the experimental measurements underestimate the Purcell factor because the shortest detectable lifetime with the use of deconvolution is ∼10 ps. Because this simulation result is the average lifetime expected, the shorter components are not resolved.

In conclusion, we have demonstrated an ultrafast spontaneous emission source with an emission speed exceeding 11 ps from a hybrid system consisting of plasmonic nanoantennas coupled to ensembles of colloidal QDs, a material particularly well suited for photonics applications. We observe large Purcell enhancements up to a factor of 880 and a 2,300-fold enhancement in the overall fluorescence combined with directional emission and high radiative quantum efficiency. The large Purcell factors are enabled by the strong field enhancement in a well-controlled sub-10-nm gap region between a silver nanocube and a Au film. The results suggest the possibility of integrating single QDs into plasmonic NPAs, expected to result in even higher Purcell factors for optimally oriented and positioned QDs, to be used as single-photon sources. If electrical injection is integrated with these structures, the NPAs could function as light-emitting diodes operating at ∼90 GHz frequencies. Furthermore, controlling the dimensions of the nanocubes and the gap thickness opens the possibility for resonances in the near infrared for spontaneous emission sources at telecommunication wavelengths.

## Methods

### Simulations

Full-wave simulations of the NPAs were performed using the commercial finite-element simulation software COMSOL Multiphysics. The scattering of a single NPA was computed based on the scattered-field formulation, in which we obtain the scattered fields by subtracting them from the analytical solution of an incident plane wave in the absence of the NPA (background field). The incidence of the excitation wave is normal to the surface. The radii of curvature of the nanocube corners were smoothed to 8 nm to obtain better agreement with the experimental scattering spectra. Moreover, the silver nanocube was surrounded by a thin 3 nm insulating poly(vinylpyrrolidinone) (PVP) shell with refractive index *n*=1.4, in agreement with the experiment. The gold film substrate of the NPA is placed on a glass substrate, which is assumed to be semi-infinite with a refractive index of *n*=1.47. The simulated scattering spectrum in Fig. 2c is calculated assuming a 7 nm polymer layer loaded in the nanogap between the cube and the gold film and with no QDs present. These polymer layers have a purely dielectric nature with an index of refraction equal to *n*=1.4. Supplementary Fig. 4 shows maps of the field enhancement in the nanogap region for an excitation wavelength of 535 nm.

To compute the spontaneous emission enhancement and the radiative quantum efficiency shown in Fig. 1d,e, the QDs were modelled as monochromatic point-dipoles emitting at the resonance of the NPA. We computed the Green's function of the system by varying the position of the dipole emitter on a discrete 15 × 15 grid placed beneath the nanocube. The surface formed by the array of dipoles was placed in the center of the spacer layer to avoid quenching as they approach the metallic parts of the plasmonic system^39^. The four-fold symmetry of the NPA was used to reduce the necessary number of simulations. The simulation domain used to compute the emissive properties of the system was similar to the domain of the scattering simulations used before. The radiative and non-radiative rates were obtained by integrating the total power radiated out of the entire domain and absorbed from the plasmonic system, respectively^44^. The dominant field component, which couples efficiently to the plasmonic resonance mode at the nanogap of the NPA, is aligned along the *z* axis. The *x* and *y* components couple weakly to the plasmonic mode in the nanogap and their contribution to the total spontaneous emission is neglected.

### Sample preparation

Ag nanocubes were colloidally synthesized using a previously described method^39^,^45^. Typically, 5 ml of ethylene glycol (EG; Aldrich, 99.8%) is heated at 150 °C for 10 min. 60 μl of 1.3 mM sodium hydrosulfide (NaSH) in EG are added to the heated EG. After 2 min, 500 μl of 3 mM hydrochloric acid (HCl) in EG and 1.25 ml of PVP (20 mg ml^−1^) in EG are added. After another 2 min, 400 μl of 0.125 M silver trifluoroacetate (AgC_2_Fe_3_O_2_) are added to the above mixture and the reaction proceeded for 2.5 h. The resulting nanocubes are centrifuged at 5,150*g* and resuspended in deionized water. The synthesis results in Ag nanocubes with side lengths of ∼70–80 nm, including a ∼3 nm residual PVP layer coating the nanocubes.

A Cr/Au (5/50 nm) film was deposited via electron beam evaporation onto a clean glass slide and, then, coated with a PAH layer with a thickness of ∼1 nm as determined using spectroscopic ellipsometry and from previous reports^37^,^39^,^42^. Core–shell CdSe/ZnS QDs (Sigma Aldrich) at a concentration of 0.1 mg ml^−1^ in toluene are spin coated onto the PAH layer at 750 r.p.m. for 5 s followed by 1,500 r.p.m. for 60 s. A diluted Ag nanocube solution (1:100) is drop cast on the sample, and the immobilized nanocubes adhere to the slightly negatively charged QDs, forming the final structure.

### Optical measurements

The optical characterization was performed using a custom-built bright-field (BF)/dark-field (DF) micro-fluorescence set-up. A × 100 DF/BF, 0.9 NA microscope objective was used for both the excitation and the collection of the scattering and fluorescence. To locate individual NPAs, an unpolarized halogen light source was used to illuminate the sample and a DF scattering image of the NPAs was captured using an electron multiplying digital camera (Hamamatsu EM-CCD, model C9100). A continuous wave *λ*_ex_=514 nm laser with a power of ∼100 μW was defocused through the objective to a ∼20-μm diameter spot on the sample. The fluorescence was collected using the same objective and imaged on the EM-CCD camera. A 550-nm longpass filter (Omega Optics) was used to reject scattered laser light. The QD fluorescence image was then overlaid with the DF image, and the NPAs that were resonant with the QDs' emission were identified. The NPA scattering and QD fluorescence spectra were characterized using a HR550 Horiba Jobin Yvon spectrometer and Symphony charge coupled device (CCD) camera. A pin-hole aperture was placed at an intermediate image plane to select light from individual NPAs.

After identification of the NPAs, fluorescence enhancement and time-resolved measurements were performed using a 535 nm pulsed laser (Ti:sapphire, 150 fs pulses at a repetition rate of 80 MHz, Coherent). The pulsed laser was focused to a diffraction-limited spot to minimize the background fluorescence from the QDs outside the NPA. The time-resolved measurements were performed at an excitation power of ∼1 kW cm^−2^, before the power dependence measurements to avoid QD bleaching. The fluorescence was detected using a fast timing avalanche photodiode (PMD, Micro Photon Device) and a time-correlated single-photon counting module (Pico-Harp 300, PicoQuant).

## Additional information

**How to cite this article:** Hoang, T. B. *et al*. Ultrafast spontaneous emission source using plasmonic nanoantennas. *Nat. Commun*. 6:7788 doi: 10.1038/ncomms8788 (2015).

## Supplementary Material

Supplementary Information Supplementary Figures 1-4, Supplementary Discussion and Supplementary References
