# Supplementary material for: Ultrafast spontaneous emission source using plasmonic nanoantennas
Source: Nat Commun. 2015 Jul 27;6:7788. doi: 10.1038/ncomms8788 (PMC4525280; doi:10.1038/ncomms8788)
Supplement: Supplementary Information — Supplementary Figures 1-4, Supplementary Discussion and Supplementary References [file ncomms8788-s1.pdf]

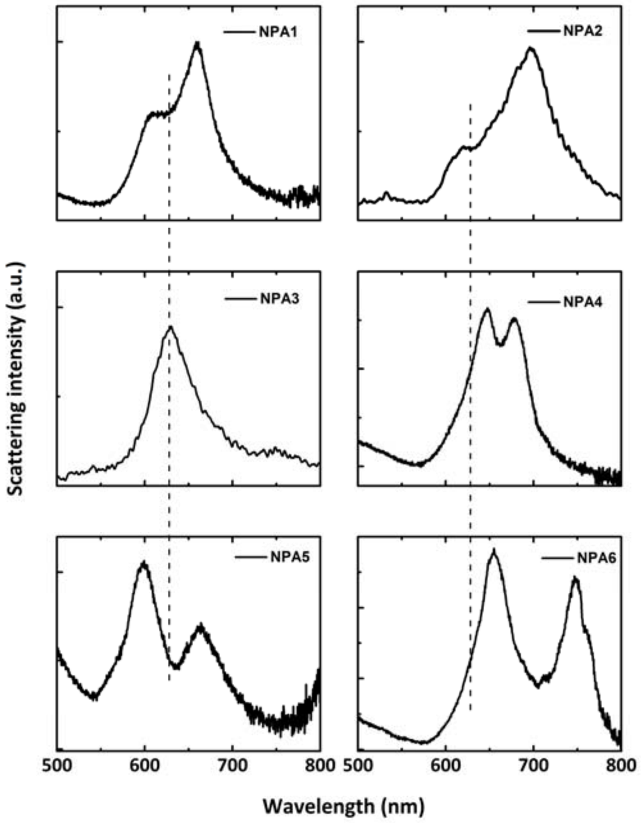

Supplementary Figure 1 | Scattering spectra from several nanopatch antennas (NPA) with the presence of quantum dots (QD) in the gap region. The QD emission wavelength is indicated by the dashed lines.

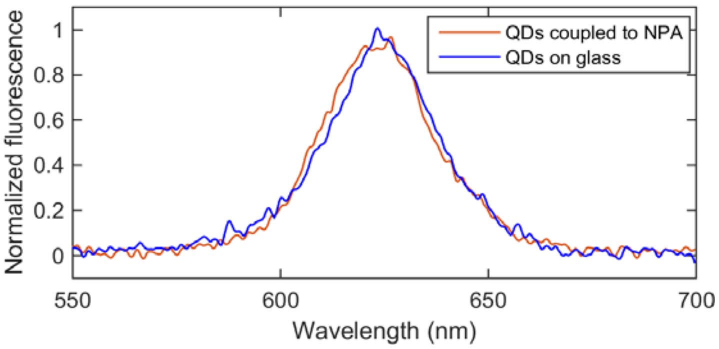

Supplementary Figure 2 | Comparison of normalized fluorescence spectrum from QDs on glass and QDs coupled to the NPA, showing that it is unmodified.

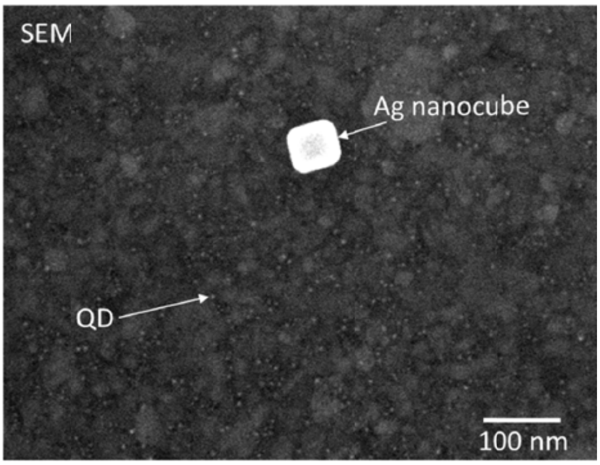

Supplementary Figure 3 | SEM image of a sample containing a single nanocube and CdSe/ZnS QDs (light small dots), similar to the samples used for the optical experiments. The image was taken within a few seconds after the area was exposed to the electron beam to avoid charging of the QDs, the nanocube, and the polyelectrolyte (PE) layers.

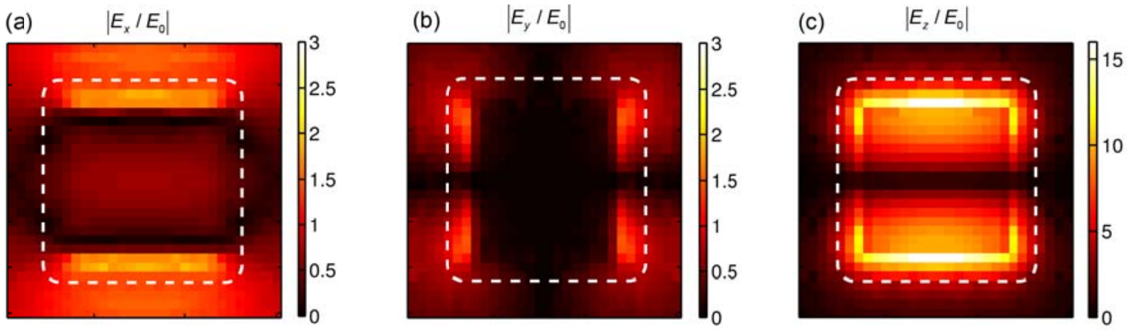

Supplementary Figure 4 | Field enhancement maps in the nanogap region for an excitation wavelength of 535 nm for the *x*, *y*, and *z* field components. The white dashed line indicates the lateral extent of the nanocube.

Supplementary Discussion

Here we discuss whether the mode splitting in the scattering spectrum observed with QDs coupled to the nanoantennas can be attributed to strong coupling. To estimate the coupling energy (Rabi splitting) between the QDs and the nanocavity mode, we use the well-known expression[1]

$$\Delta E_0 = \hbar g = \vec{\mu} \cdot \vec{E}_{vac} \quad (1)$$

where ΔE is the coupling energy, g is the coupling rate, μ is the dipole moment for a single QD, and E_{vac} is the vacuum electric field. The dipole moment for a single epitaxial QD was found by

Yoshie et al.[2] to be μ = 29 D. Here we make the approximation that the colloidal QDs have a transition dipole moment of a similar magnitude. This assumption is supported by the similar magnitude of the absorption cross-section that has been measured for colloidal QDs[3] ($\sigma = 1.1 \times 10^{-14}$ cm^2) and for epitaxial QDs[4] ($\sigma = 4 \times 10^{-15}$ cm^2). The vacuum electric field in a cavity of mode volume V_m is[2]

$$E_{vac} = \sqrt{\frac{hc}{2\lambda V_m n^2 \varepsilon_0}} \qquad (2)$$

where λ is the wavelength of light, n is the refractive index of the cavity region, and ε_0 is the vacuum permittivity. The cavity mode volume is estimated based on the geometrical dimensions of the nanocube cavity given by V_m = 75 nm × 75 nm × 10 nm = 5.6×10^{-5} μm^3 with a refractive index of n = 1.5. This estimate of the cavity mode volume is consistent with the volume obtained from the Purcell factor formula $F = 4/3\pi^2(\lambda/n)^3(Q/V_m)$, which gives V_m = 2.3×10^{-5} μm^3. From Eqs. (1) and (2) and the geometrical volume, we find a single QD coupling energy of $\Delta E_0 = 7$ meV. As the number of coupled dipoles N is increased, the coupling energy follows the relationship $\Delta E = \sqrt{N}\Delta E_0$. Based on transmission electron microscopy (TEM) analysis, a typical nanocube is coupled to ~10 QDs. Hence, the typical coupling energy we expect is $\Delta E = 22$ meV. At λ = 650 nm, this corresponds to a splitting of $\Delta\lambda = 7$ nm, which is much smaller than the linewidth of the cavity mode (~50 nm corresponding to $\Delta E_{cav} = 150$ meV) and the linewidth of the QD ensemble exciton absorption (~35 nm corresponding to $\Delta E_{QD} = 100$ meV). Therefore, the conditions for strong coupling are not satisfied in our experiment and the system operates in the weak coupling regime, making application of the Purcell effect valid.

To reach the regime of strong coupling in the future, such that $\Delta E \geq \Delta E_{cav}$, ~450 QDs would need to be coupled to a single nanocavity. The volume of the nanocavity dimensions allow for a maximum of only ~150 QDs to be coupled assuming a close-packed single layer. Therefore, we believe reaching strong coupling may not be possible with the current geometry and QD materials.

As experimental verification that our system is in the weak coupling regime, we find that the emission spectrum of the QDs is not modified when coupled to the NPA, as seen in Supplementary Figure 2. This is in contrast to the behavior observed, for example, for J-aggregates coupled to a plasmonic structure, in which the fluorescence spectrum is strongly modified under conditions of strong coupling.[5]

Supplementary References

1. Kavokin, A., Baumberg, J. J., Malpuech, G. & Laussy, F. P. *Microcavities*. (OUP Oxford, 2011).

2. Yoshie, T. *et al.* Vacuum Rabi splitting with a single quantum dot in a photonic crystal nanocavity. *Nature* **432,** 200–203 (2004).

3. Leatherdale, C. a., Woo, W.-K., Mikulec, F. V. & Bawendi, M. G. On the Absorption Cross Section of CdSe Nanocrystal Quantum Dots. *J. Phys. Chem. B* **106,** 7619–7622 (2002).

4. Osborne, S. W. *et al.* Optical absorption cross section of quantum dots. *J. Phys. Condens. Matter* **16,** S3749–S3756 (2004).

5. Schlather, A. E., Large, N., Urban, A. S., Nordlander, P. & Halas, N. J. Near-field mediated plexcitonic coupling and giant Rabi splitting in individual metallic dimers. *Nano Lett.* **13,** 3281–3286 (2013).
